# Effects of Pup Separation on Stress Response in Postpartum Female Rats

**DOI:** 10.3390/ijms18071370

**Published:** 2017-06-27

**Authors:** Manu Kalyani, Phyllis Callahan, James M. Janik, Haifei Shi

**Affiliations:** Department of Biology, Miami University, Oxford, OH 45056, USA; kalyanm@miamioh.edu (M.K.); callahp@miamioh.edu (P.C.); janikjm@miamioh.edu (J.M.J.)

**Keywords:** lactation, prolactin, prolactin receptor, HPA axis, corticosterone, restraint stress

## Abstract

There is a complex collection of neuroendocrine function during the postpartum period. Prolactin (PRL) released by suckling stimulus and its PRL receptors (PRL-R) in the central nervous system (CNS) are involved in hyporesponsiveness of the hypothalamic-pituitary-adrenal (HPA) axis in lactating mammals including rodents and humans. It is not clear how long it takes to reestablish the attenuated HPA axis activity of lactating rats to a pre-pregnancy state after pup separation. We first tested the hypothesis that HPA axis activity in response to an acute stress in postpartum rats would return to a pre-pregnancy state after pup separation. Restraint stress for 30 min was performed at the end of pup separation as an acute stressor. Plasma levels of corticosterone (CORT) were measured following restraint stress or no-stress (control) in virgin rats and postpartum rats housed with their pups or with pup removal for different periods of time of one hour, 24 h, or eight days. We then tested the hypothesis that circulating PRL level and CNS PRL-R gene expression were involved in mediating the acute stress response in postpartum rats. Plasma levels of PRL and PRL-R mRNA levels in the choroid plexus of the CNS were determined in both no-stress and stress, virgin rats, and postpartum rats housed with their pups or with pup removal for various periods, and their correlation with plasma CORT levels was assessed. The results demonstrated that PRL levels declined to virgin state in all postpartum rats separated from their pups, including the dams with one-hour pup separation. Stress-induced HPA activity dampened in lactating rats housed with pups, and returned to the pre-pregnancy state after 24 h of pup separation when both circulating PRL level and CNS PRL-R expression were restored to a pre-pregnancy state. Additionally, basal plasma CORT and CNS PRL-R expression were significantly correlated in rats with various pup status. This study suggested that stress-induced HPA activation occurred when PRL-R expression was similar to the level of virgin females, indicating that PRL-R upregulation contributes to an attenuated HPA response to acute stress. Understanding neuroendocrine responses to stress during the postpartum period is critical to understand postpartum-related neuropsychiatric illnesses and to maintain mental health in postpartum women.

## 1. Introduction

According to an analysis of 2012 Department of Labor survey data, conducted in 2015 by researchers at Abt Associates (Cambridge, MA) and published online at “In These Times” [1], 23% of new mothers surveyed returned to work within only two weeks of giving birth. Numerous physiological changes occur during the postpartum period at the onset of lactation in mammals [2,3]. Prolactin (PRL), a 23-kDa polypeptide hormone synthesized in, and secreted from, anterior pituitary lactotrophs, plays important roles in the initiation and maintenance of lactation in mammals [4,5,6]. Suckling is the most potent stimulus for PRL release from the anterior pituitary [5,7,8,9]. Increased circulating PRL level during lactation leads to an upregulation of PRL receptor (PRL-R) in several regions of the central nervous system (CNS), including the choroid plexus, preoptic area, and hypothalamic nuclei [10,11,12]. Circulating PRL gains access to the brain through a PRL-R mediated transport via choroid plexus cells [13], and PRL mediates its action through the PRL-R in the CNS [13,14,15,16,17].

In addition to its roles in regulating reproductive functions, PRL attenuates the response to stress [3,18,19]. During lactation, there is a chronic increase in basal plasma glucocorticoid levels [20,21,22], along with reduced expression of corticotropin releasing hormone (CRH) within the paraventricular nucleus (PVN) [23,24,25]. Additionally, the responsiveness of the hypothalamic-pituitary-adrenal (HPA) axis to stress is reduced during lactation, indicated by blunted releases of adrenocorticotropic hormone (ACTH) and glucocorticoids in rodents [3,20,25] and humans [26]. Elevated levels of PRL along with increased PRL-R expression in the PVN reduce HPA axis activity in lactating females [3,23,27]. Furthermore, when virgin female rats receive a lateral cerebroventricle infusion (icv) of PRL, their response is similar to the response that occurs during the hyperprolactinemic state of lactation, i.e., increased CNS PRL produces an attenuation of stress-induced ACTH secretion [19]. Finally, icv of an antisense PRL-R probe in lactating rats results in an increase in the stress-induced ACTH response [3]. These studies indicate that PRL is an important neuroendocrine hormone involved in attenuation of HPA axis activation during lactation [25,27]. It is important to note that these studies have been conducted during periods of chronic hyperprolactinemia, i.e., either during lactation or when PRL levels are experimentally elevated for a prolonged period of time.

Although a substantial literature exists documenting a lactation-related hyporesponsive state of HPA activity, it is not clear if pup separation and thus temporary reverse hyperprolactinemia would recover HPA activity to the pre-pregnancy, virgin state. We tested the hypothesis that HPA axis activity would return to a pre-pregnancy state after pup separation, and that circulating PRL level and CNS PRL-R gene expression are involved in mediating the acute stress response. Additionally, the duration of pup separation required to recover the attenuated HPA activity to a pre-pregnancy state is unknown. The purpose of this study was (1) to investigate the relevance of PRL and its receptor in mediating the stress response in lactating rats, and (2) to determine the duration of pup separation required to reestablish the dampened HPA responsiveness to an acute stress during the postpartum period. Restraint stress was used as an acute stressor, as it is one of the most commonly employed procedures to induce stress-related behavioral, biochemical and physiological changes in laboratory animals [28]. Basal and stress levels of corticosterone (CORT) and PRL, as well as PRL-R mRNA levels in the choroid plexus, were determined before and 30 min after the termination of stress in virgin rats, postpartum mid-lactating rats housed with their pups, or postpartum rats with pup removal for different periods of time. The results demonstrated that the stress-induced response of the HPA activity returned to the pre-pregnancy state after 24 h of pup separation, when the circulating PRL and CNS PRL expression were restored to a pre-pregnancy state.

## 2. Results

### 2.1. Circulating CORT Levels of Virgin and Lactating Rats with or without Pup Separation Following No-Stress or an Acute Stress

**Figure 1:**
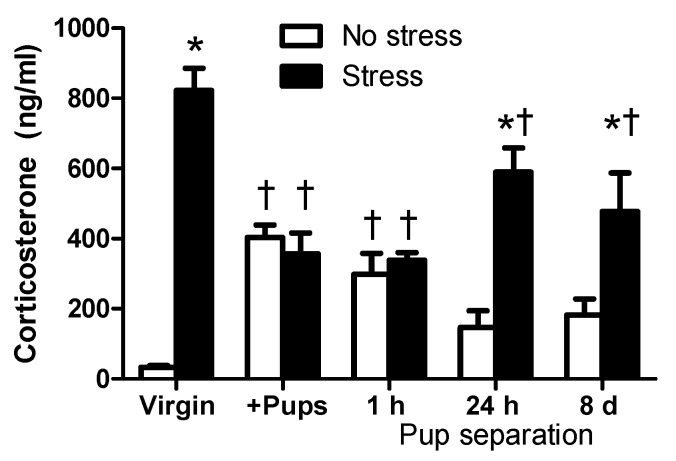
The effects of pup status on circulating corticosterone (CORT) levels following restraint stress. No-stress rats (white bars) were left undisturbed in their home cage for 60 min prior to sacrifice. Stressed rats (black bars) were subjected to a 30 min restraint stress and then returned to their home cage for 30 min before sacrifice. Virgin female rats (no-stress: *n* = 8, stress: *n* = 11), postpartum females housed with their pups (+Pups; no-stress: *n* = 11, stress: *n* = 11) or separated from their pups (Pup separation) for one hour (1 h Pup separation; no-stress: *n* = 9, stress: *n* = 8), 24 h (24 h Pup separation; no-stress: *n* = 11, stress: *n* = 11), or eight days (8 d Pup separation; no-stress: *n* = 10, stress: *n* = 11). Trunk blood was collected at the time of sacrifice, and plasma samples were measured for circulating CORT levels. * Significantly different from “No-stress” females within the same group. † Significantly different from the virgin females with the same stress status. Values are means ± SEM.

The circulating CORT levels of virgin females following an acute restraint stress were significantly higher than that of no-stress virgin rats (*t* = 8.817, *p* < 0.001; Figure 1). In contrast, circulating CORT levels were not different between stress and no-stress groups of the postpartum females housed with their pups or separated from their pups for one hour. Additionally, stress increased circulating CORT levels in the postpartum females that were separated from their pups for 24 h (*t* = 5.400, *p* < 0.001) or eight days (*t* = 3.334, *p* < 0.01; Figure 1). It is noteworthy that, among the no-stress groups, the CORT levels of virgin females were significantly lower than those of the postpartum females housed with their pups (*t* = 4.718, *p* < 0.001) and the females separated from their pups for one hour (*t* = 2.86, *p* < 0.05), but was not significantly different compared with postpartum females after a longer period of pup separation of either 24 h (*t* = 1.276, *p* > 0.05) or eight days (*t* = 1.612, *p* > 0.05). Post hoc tests revealed the main effects of stress treatment (F_(1,89)_ = 60.79, *p* < 0.0001) but not pup status (F_(4,89)_ = 1.00, *p* = 0.4105). There was significant interaction between pup status and stress for CORT levels (F_(4,89)_ = 14.67, *p* < 0.0001). In summary, basal CORT levels were higher in postpartum female rodents housed with their pups, and they declined as the duration of pup separation increased. Stress-induced higher circulating CORT levels were seen in virgin females and in dams with pup separation for 24 h or eight days whose CORT levels of no-stress groups were not significantly different from no-stress virgin rats.

### 2.2. Circulating PRL Levels of Virgin and Lactating Rats with or without Pup Separation Following No-Stress or an Acute Stress

**Figure 2:**
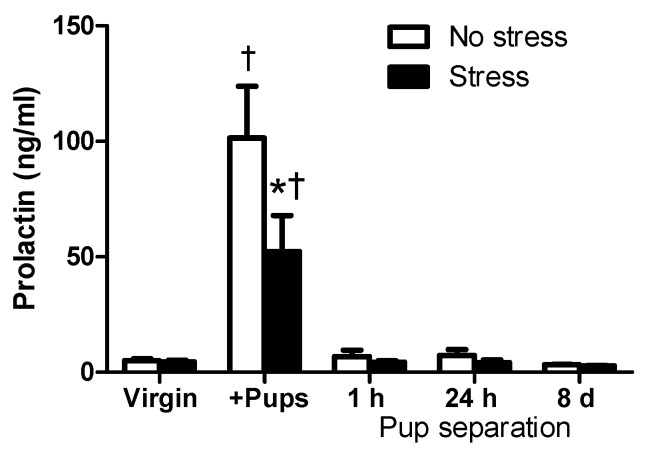
The effects of pup status on circulating prolactin (PRL) levels following restraint stress. No-stress rats (white bars) were left undisturbed in their home cage for 60 min prior to sacrifice. Stressed rats (black bars) were subjected to 30 min of restraint stress and then returned to their home cage for 30 min before sacrifice. Virgin female rats (no-stress: *n* = 7, stress: *n* = 8), postpartum females housed with their pups (+Pups; no-stress: *n* = 10, stress: *n* = 9) or separated from their pups (Pup separation) for one hour (1 h Pup separation; no-stress: *n* = 8, stress: *n* = 9), 24 h (24 h Pup separation; no-stress: *n* = 11, stress: *n* = 11), or eight days (8 d Pup separation; no-stress: *n* = 8, stress: *n* = 5). Trunk blood was collected at the time of sacrifice, and plasma samples were measured for circulating PRL levels. * Significantly different from “No-stress” females within the same group. † Significantly different from the virgin females with the same stress status. Values are means ± SEM.

Consistent with previous studies [8,11], circulating PRL levels of the postpartum females housed with their pups were significantly higher than the PRL levels of the virgin female rats, due to the stimulation from suckling, between no-stress groups (*t* = 6.782, *p* < 0.001) and between stress groups (*t* = 3.416, *p* < 0.001; Figure 2). In contrast, PRL levels returned to the same levels as the virgin females in all pup separation groups, regardless of the time of separation. Additionally, 30 min of restraint stress reduced PRL levels of postpartum rats housed with pups (*t* = 3.701, *p* < 0.01), but had no effect on PRL levels of virgin rats or postpartum rats that were separated from their pups for one hour, 24 h, or eight days (Figure 2). Post hoc tests revealed the main effects of pup status (F_(4,76)_ = 20.49, *p* < 0.0001), but not stress (F_(1,76)_ = 3.03, *p* = 0.0857). The interaction between pup status and stress was not significant for circulating PRL levels (F_(4,76)_ = 2.22, *p* = 0.0747). In summary, PRL levels were higher in lactating rats housed with their pups, comparing with virgin rats and dams separated from their pups.

### 2.3. PRL-R mRNA Levels of Virgin and Lactating Rats with or without Pup Separation Following No-Stress or an Acute Stress

**Figure 3:**
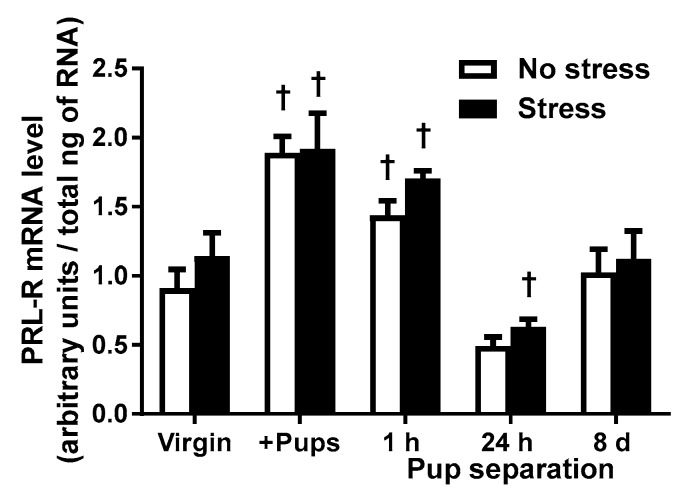
The effects of pup status on prolactin receptor (PRL-R) mRNA levels following restraint stress. No-stress rats (white bars) were left undisturbed in their home cage for 60 min prior to sacrifice. Stressed rats (black bars) were subjected to 30 min of restraint stress and then returned to their home cage for 30 min before sacrifice. The choroid plexus tissue from two rats was pooled and samples were run in triplicate. Virgin female rats (no-stress: *n* = 6 stress: *n* = 6), postpartum females housed with their pups (+Pups; no-stress: *n* = 6, stress: *n* = 6) or separated from their pups (Pup separation) for 1 h (1 h Pup separation; no-stress: *n* = 4, stress: *n* = 4), 24 h (24 h Pup separation; no-stress: *n* = 8, stress: *n* = 8), or eight days (8 d Pup separation; no-stress: *n* = 6, stress: *n* = 6). PRL-R mRNA levels in the choroid plexus are expressed as a percent of the non-stressed, virgin females. † Significantly different from the virgin females with the same stress status. Values are means ± SEM.

The PRL-R mRNA levels in the choroid plexus were significantly higher in postpartum females housed with their pups than their virgin female counterparts, between no-stress groups (*t* = 4.674, *p* < 0.001) and stress groups (*t* = 3.717, *p* < 0.01), likely due to high circulating PRL levels that occur during suckling. PRL-R mRNA levels of postpartum stressed females remained significantly higher than those of virgin females even after one-hour pup separation (*t* = 2.409, *p* < 0.05; Figure 3). After 24 h of pup separation, PRL-R mRNA levels of the stressed rats were significantly reduced comparing with the levels of the stressed virgin females (*t* = 2.604, *p* < 0.05), although the PRL-R mRNA levels were not significantly different between no-stress virgin rats and postpartum rats with 24-h pup separation (*t* = 2.145, *p* > 0.05). After eight days of pup separation, PRL-R mRNA levels were similar to the levels of virgin females in both no-stress and stress groups (*p* > 0.05). Acute stress did not produce any change in PRL-R mRNA levels in virgin females or any postpartum group (Figure 3). Post hoc tests revealed significant main effects of pup status (F_(4,50)_ = 23.57; *p* < 0.0001), without significant main effects of stress (F_(1,50)_ = 2.54, *p* = 0.1173), on the PRL-R mRNA levels in the choroid plexus. The interaction effect between pup status and stress was not significant for PRL-R expression (F_(4,50)_ = 0.20, *p* = 0.9354). In summary, the increased PRL-R expression was seen in lactating rats housed with their pups and in dams after one-hour pup separation. PRL-R expression decreased to pre-pregnancy level after 24-h or eight-day pup separation in postpartum rats.

### 2.4. Correlation Among CORT, PRL, and PRL-R mRNA Levels of Virgin and Lactating Rats with or without Pup Separation Following No-Stress or an Acute Stress

**Figure 4:**
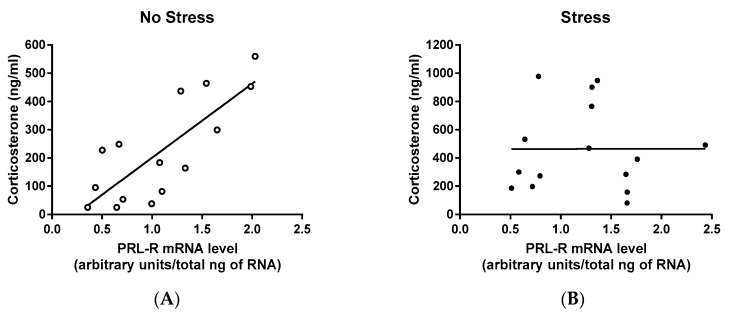

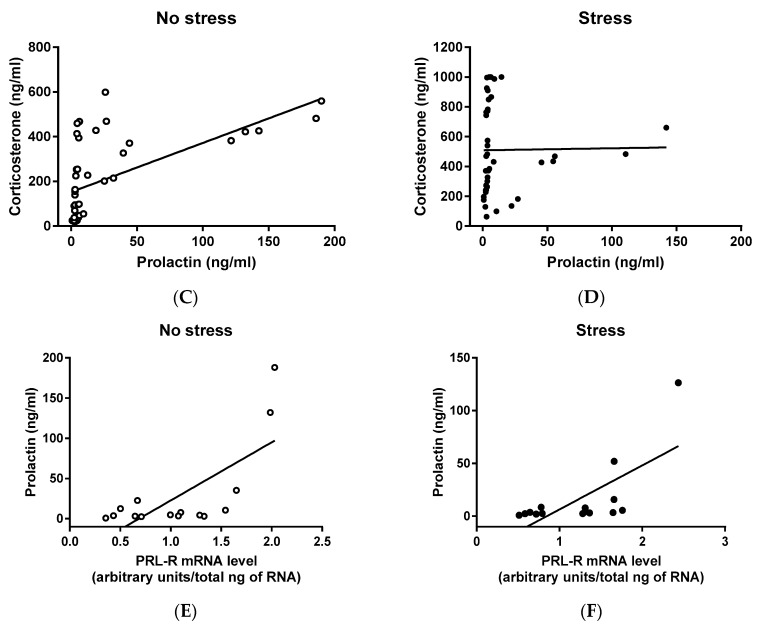
Correlation between circulating corticosterone (CORT) levels and the central nervous system (CNS) prolactin receptor (PRL-R) mRNA levels (**A**,**B**), between circulating CORT and prolactin (PRL) levels (**C**,**D**), and between circulating PRL and CNS PRL-R mRNA levels (**E**,**F**) of virgin and lactating rats with or without pup separation following no-stress or an acute stress. No-stress rats were left undisturbed in their home cage for 60 min prior to sacrifice. Stressed rats were subjected to 30 min of restraint stress and then returned to their home cage for 30 min before sacrifice. The only significant correlation detected was between circulating CORT and CNS PRL-R expression in no-stress rats (**A**).

The correlation among circulating levels of CORT and PRL and gene expression of PRL-R were analyzed to reveal a potential relationship among these variables. There was a significant correlation between plasma CORT and CNS PRL-R expression in no-stress rats (*r*^2^ = 0.843, *p* = 0.036; Figure 4A), but not in stressed rats (*r*^2^ = 0.643, *p* = 0.121; Figure 4B). The correlation between plasma levels of CORT and PRL was not significant among either no-stress rats (*r*^2^ = 0.755, *p* = 0.0701; Figure 4C) or stressed rats (*r*^2^ = 0.441, *p* = 0.229; Figure 4D). Additionally, there was no significant correlation between plasma PRL level and CNS PRL-R expression among no-stress groups (*r*^2^ = 0.771, *p* = 0.063; Figure 4E) or stress groups (*r*^2^ = 0.673, *p* = 0.106; Figure 4F). In summary, the only significant correlation observed from this study was between plasma levels of CORT and CNS PRL-R expression in no-stress rats.

## 3. Discussion

More women than before return to work and face stressful conditions [29] after a decreasing period of maternity leave, especially in countries that do not offer guaranteed paid leave for women after childbirth, including the United States [30]. As the numbers of dual-career couples and single working parents increase, this trend is expected to keep rising. Although it is known that chronic hyperprolactinemic conditions during lactation inhibit HPA axis activation in response to stress [25,31], which is important for maintaining maternal mental health, the duration of pup separation and, thus, temporary reversed hyperprolactinemia for various periods of time required to recover HPA axis activation is unclear, however. The present study began to understand if (1) the lactation hormone PRL and its receptor PRL-R in the CNS, and (2) the duration of pup separation were involved in mediating stress response using virgin and postpartum lactating rodent models. Restraint stress was used as an acute stressor, as it is one of the most commonly employed procedures to induce stress-related behavioral and physiological changes in laboratory animals [28]. It is noteworthy that no-stress dams that were separated from their pups for various periods did not increase their circulating CORT levels (Figure 1), suggesting that pup removal alone did not activate the HPA axis. Therefore, pup separation is not a robust stress inducer compared with the restraint stress.

Circulating CORT levels under rest and stressed conditions were measured as an indicator of HPA axis activity [32] in virgin and postpartum rats. Measuring other components of the HPA axis, such as CRH within the PVN and circulating ACTH levels, would provide a comprehensive understanding of the HPA axis activity in responding to pup separation in postpartum rats. HPA response to stress underwent distinct changes during lactation and following pup removal [24,25,33]. Current results demonstrated that HPA axis activity, under both no-stress and stress conditions, were different during the postpartum period from the pre-pregnancy, virgin state. To our knowledge, this is the first study demonstrating basal and stress-induced CORT levels in postpartum females after various durations of pup separation. Consistent with the literature that basal CORT levels are higher in postpartum female rodents housed with their pups than basal CORT levels of virgin females [20,21], the present study found that CORT levels under no-stress condition were elevated in postpartum females housed with their pups. Additionally, CORT levels declined over time as the duration of pup separation increased. These results suggest that the postpartum lactation period is metabolically demanding and stressful [20,34]. In agreement with previous reports [21,24,35], stress-induced activation of the HPA axis seen in virgin rats, indicated by significantly higher circulating CORT levels than their no-stress counterparts, was diminished in lactating rats that remained housed with their pups or separated from their pups for one hour, and was restored after pup separation for 24 h or eight days when CORT levels and PRL-R expression of the no-stress groups were no longer significantly different from the no-stress virgin rats (Figure 1 and Figure 3). Therefore, stress-induced activation of the HPA axis was blunted during the postpartum period, but only when dams were housed with their pups or briefly separated for one hour. One possible explanation for the blunted HPA axis activation following stress is that sustained, high levels of PRL and CORT, as occurs during lactation, attenuate the HPA axis response to stress [23,36].

As expected, the results from the present study indicated that PRL levels were low in virgin rats, PRL levels were high in lactating rats housed with their pups as a result of suckling, and PRL levels declined to pre-pregnancy values as early as one hour after pups were removed from the dams (Figure 2). In contrast, the increased PRL-R expression in lactating rats was kept elevated after one-hour pup separation, declined after 24-h pup separation, albeit not significantly, and returned to pre-pregnancy levels by eight days of pup separation in postpartum rats (Figure 3). In addition to its important role in the initiation and maintenance of lactation [5,19], PRL, similar to the glucocorticoids, is considered a stress hormone that inhibits HPA axis activity during lactation [4] or under chronic hyperprolactinemic conditions [25,31]. Elevated circulating PRL levels following stress, along with increased PRL-R mRNA levels in the choroid plexus, have been reported in the literature [37,38]. The stress-induced increase in circulating PRL levels causes upregulation of PRL-R in the choroid plexus and the PVN of the hypothalamus, eliciting the transport of circulating PRL into the brain via PRL-R in the choroid plexus [13,14,15] and acting on PRL-R in the PVN [39], respectively. PRL in the brain seems to exert a protective effect; icv administration of PRL protects against stress-induced hypoglycemia, ulcerogenesis [37], and hyperthermia [40]. Additionally, there is an interaction between the activation of the HPA axis and PRL release. Pharmacological antagonists of CRH receptors attenuate the stress-induced release of both CORT and PRL, suggesting that the activation of the HPA axis may stimulate PRL release [41]. Acute increases in PRL levels activated the ERK/MAPK pathway in the CRH neurons in the PVN suggesting PRL may stimulate the HPA axis activity initially [39], but inhibit it under more chronic conditions [27]. Other neurohormones could regulate PRL levels during stress. Oxytocin, for example, is closely linked to the HPA axis due to its expression in the PVN [42]. Additionally, oxytocin secretion into the blood increases in response to various stressors [43]. Thus, oxytocin is considered as a stress hormone. Furthermore, oxytocin is a stimulating factor for PRL secretion in female rats [44]. Whether or not increased expression of oxytocin within the hypothalamus and/or circulating oxytocin levels contributes to the increased PRL levels in stressed rats will be studied in the future.

In the current study, stress did not increase circulating PRL levels or PRL-R mRNA levels in either virgin females or any postpartum group (Figure 3). While stress has been reported to increase circulating PRL levels and PRL-R mRNA levels in the choroid plexus [37,38], there are several important differences between the current studies and previous reports. First, in the current study, animals were subjected to 30 min of acute restraint stress, whereas Fujikawa et al. [38] subjected animals to seven hours of restraint stress in water, which was much longer in duration and a more intense stimulus than the stressor employed in the current study. Additionally, male rats were used in the previous studies [14,37], and sex differences may contribute to the lack of consistency in findings. In the current study, HPA axis activation was not likely due to an increased level of PRL in the CNS that stimulates CRH neurons because PRL-R mRNA expression did not differ between no-stress and stress groups with the same pup status. There are other factors that may contribute to the regulation of HPA axis activity during lactation. For example, arginine vasopressin (AVP) has been co-localized with CRH in the PVN [23,45,46], indicating that AVP may increase CRH neuronal activity and ACTH secretion, which would stimulate CORT release [23].

There are two noteworthy points. One point is that PRL-R expression showed delayed change comparing with PRL levels. The hyperprolactinemia that occurs during lactation is due to the neural stimulus of suckling and inhibitory regulation of dopaminergic neurons [12,47], an acutely regulated process. The half-life for mRNA is approximately three hours [48], therefore, one hour of pup separation was not enough time for CNS PRL-R mRNA levels to return to the pre-pregnancy state. After 24 h or eight days of separation, however, PRL-R mRNA levels were reduced and then returned to the pre-pregnancy state eight days after lactation was terminated. In agreement with Grattan et al. [12], postpartum females housed with their pups had high PRL-R mRNA levels, likely due to high circulating PRL levels that occur during suckling. After one hour of pup separation, when PRL levels had significantly decreased, PRL-R mRNA levels remained significantly higher than virgin females (Figure 2). The other point is that there was a significant correlation between the CORT levels and PRL-R, instead of PRL, suggesting that basal PRL uptake into the brain is critical for activation of the HPA axis by acute stress. Stress-stimulated CORT level increases occurred only when PRL-R expression was similar to its expression in the virgin females, indicating that PRL-R upregulation that occurs during chronic hyperprolactinemia, instead of temporary changes in circulating PRL levels, contributes to an attenuated HPA axis response to stress.

## 4. Materials and Methods

### 4.1. Animals

Adult male and virgin female Sprague-Dawley rats (200–225 g; 10–12 weeks old; Harlan Laboratories, Indianapolis, IN, USA) were housed in a clean, stress-free environment under controlled lighting (12 h light:dark; lights on at 0600 and lights off at 1800) and temperature (21 °C). Food and water were provided ad libitum. A group of females was mated with adult male rats and daily vaginal smears were taken at 0800–0900. The presence of sperm in the smear was designated as day 0 of pregnancy. The litter size was culled to eight pups on postpartum day 3. A group of age-matched virgin females and mid-lactating (10–14 days postpartum) rats [49] were used in this study. During mid-lactation, PRL-R expression is most sensitive to the suckling stimulus [8]. Miami University Institutional Animal Care and Use Committee approved all procedures (project No. 882) in accordance with the National Institutes of Health Guidelines for the Care and Use of Laboratory Animals.

### 4.2. Restraint Stress and Sample Collection

CORT levels and HPA axis activity in rodents reach nadir at the beginning of the light phase [50]. Pup separation started at the beginning of the light cycle at 0630. Stress tests or no-stress procedures started at the end of pup separation and were performed between 0730 and 0930 to avoid any effects of circadian variation in hormone levels. There were five groups: virgin female rats, postpartum females housed with their pups, or separated from their pups (Pup separation) for one hour, 24 h, or eight days.

Animals that were subjected to stress were placed in a plexiglass restraint chamber for 30 min, returned to their home cage, and sacrificed 30 min later. Control (no-stress) rats remained undisturbed in their home cage for 60 min before sacrifice. Therefore, trunk blood was collected in heparinized (1000 U/mL) tubes and the plasma was collected and kept frozen at −20 °C until subjected to radioimmunoassay. Immediately after sacrifice, the brain was removed, placed in ice-cold saline, and blocked at the olfactory bulb, rostrally, and the mammillary bodies, caudally. The lateral ventricles were exposed, and the choroid plexus with highest density of long form PRL-R was collected and stored at −80 °C until assayed. Choroid plexus tissue from two rats was pooled to ensure sufficient levels of mRNA for quantitative real-time PCR (qRT-PCR).

### 4.3. Hormone Assays

Plasma CORT levels were measured in triplicate samples using double antibody radioimmunoassay kits following the manufacturer’s instructions (MP Biomedicals, Santa Ana, CA, USA). Plasma PRL levels were measured in triplicate samples using double antibody radioimmunoassay reagents provided by the National Hormone and Peptide Program (NHPP and A. F. Parlow). Na^125^I labeled PRL was purchased from Perkin Elmer (Waltham, MA, USA). Plasma CORT and PRL concentrations are expressed as ng/mL.

### 4.4. Quantitative RT-PCR

Total RNA was isolated from the choroid plexus using the Mini RNeasy kit (Qiagen, Valencia, CA, USA) per the manufacturer’s instructions for rats. Briefly, the tissue was sonicated (Branson Sonifier-250, Danbury, CT, USA) in 350 µL of lysis buffer (Qiagen, Valencia, CA, USA). Turbo DNAse (Ambion, TX, USA) was used to treat the RNA following the manufacturer’s instructions. RNA was reverse-transcribed under the following conditions: 0.5 µg of RNA, 2 µL of random hexamers (0.2 µg/µL; Promega, Madison, WI, USA), and nuclease-free H_2_O were added to bring the total reaction volume to 12 µL. This mixture was heated at 70 °C for 10 min, and then cooled at 4 °C. Promega RT buffer (1×), dNTPs (667 µM), MgCl_2_ (3.3 mM), nuclease-free H_2_O up to 30 µL and one unit of Improm II reverse transcriptase enzyme (Promega, WI, USA) were added to tubes that were held at 4 °C. Negative controls contained all reaction components except for the RT enzyme. The reaction was held at 25 °C for 5 min, heated to 42 °C for one hour, and a final enzyme deactivation at 70 °C for 15 min. All cDNA was purified using a Qiaquick nucleotide removal kit (Qiagen, Valencia, CA, USA) and eluted from the column with 60 µL of the elution buffer.

Quantitative RT-PCR was run using the standard curve method [51]. Briefly, cDNA from each analyzed sample was pooled to generate a five-point standard curve. The first point was a 1:1 dilution of the pool in nuclease-free H_2_O; the remaining standards were generated by serial dilution (four serial 1:5 dilutions for a total of five standards). Samples were diluted 1:50 in nuclease-free H_2_O. The standards were run in duplicate and the samples were run in triplicate. Each sample had a negative control, i.e., no RT enzyme was added. The total reaction volume (20 µL), contained: Q quantitect SYBR mix (Qiagen, Valencia, CA, USA), additional SYBR green, MgCl_2_, and PRL-R primers (Accession: XM_017590635; forward: 5′-CTGGGCAGTGGCTTTGAAG-3′, reverse: 5′-CCAAGGCACTCAGCAGCTCT-3′). Values obtained were normalized to constitutive house-keeping gene β actin (Accession: NM_031144; forward: 5′-AGATGACCCAGATCATGTTTGAGA-3′, reverse: 5′-ACCAGAGGCATACAGGGACAA-3′). All reactions were conducted using a Rotorgene 3000 (Corbett Life Science, Sydney, Australia).

### 4.5. Statistical Analysis

Data were analyzed using Prism Statistical Software (Version 5, GraphPad Software, Inc., La Jolla, CA, USA). Data were expressed as mean ± SEM. For each parameter, a two-way analysis of variance (ANOVA; rat group × stress treatment) followed by Bonferroni’s multiple comparison test was performed. Post hoc multiple comparison tests were carried out with computing the 95% confidence interval (CI) of the difference between two group means and the test was considered statistically significant if this CI did not include zero. Additionally, correlations between plasma CORT and PRL levels, plasma CORT and CNS PRL-R mRNA levels, and plasma PRL and CNS PRL-R mRNA levels were performed using linear regression, *r*^2^ was calculated to indicate goodness of fit, and *p* values were calculated to indicate significance of correlation (Prism). A test with *p*-values less than 0.05 (i.e., *p* < 0.05) was considered statistically significant.

## 5. Conclusions

The hormonal milieu is substantially different from other physiological states in lactating females. This study demonstrated that the HPA axis response to stress was dampened in lactating postpartum females and females with short-term one-hour pup separation, but was restored to the pre-pregnancy state after 24 h or eight days of pup separation. Understanding the neuroendocrine mechanisms involved in regulating stress response in postpartum females is important in maintaining mental health and treating stress-related disorders in postpartum women.

## Abbreviations

icv	Cerebroventricle infusion
ACTH	Adrenocorticotropic hormone
CNS	Central nervous system
CORT	Corticosterone
CRH	Corticotropin releasing hormone
HPA	Hypothalamic-pituitary-adrenal
PRL	Prolactin
PRL-R	Prolactin receptor
PVN	Paraventricular nucleus
qRT-PCR	Quantitative real-time PCR
